# Genetic Advancements in Infantile Epileptic Spasms Syndrome and Opportunities for Precision Medicine

**DOI:** 10.3390/genes15030266

**Published:** 2024-02-21

**Authors:** Hannah E. Snyder, Puneet Jain, Rajesh RamachandranNair, Kevin C. Jones, Robyn Whitney

**Affiliations:** 1 Division of Neurology, Department of Paediatrics, McMaster University, Hamilton, ON L8N 3Z5, Canadarnair@mcmaster.ca (R.R.);; 2 Division of Neurology, Department of Paediatrics, The Hospital for Sick Children, University of Toronto, Toronto, ON M5G 1E8, Canada

**Keywords:** developmental and epileptic encephalopathy (DEE), epilepsy, genetics, infantile spasms, epileptic spasms, precision medicine, West syndrome

## Abstract

Infantile epileptic spasms syndrome (IESS) is a devastating developmental epileptic encephalopathy (DEE) consisting of epileptic spasms, as well as one or both of developmental regression or stagnation and hypsarrhythmia on EEG. A myriad of aetiologies are associated with the development of IESS; broadly, 60% of cases are thought to be structural, metabolic or infectious in nature, with the remainder genetic or of unknown cause. Epilepsy genetics is a growing field, and over 28 copy number variants and 70 single gene pathogenic variants related to IESS have been discovered to date. While not exhaustive, some of the most commonly reported genetic aetiologies include trisomy 21 and pathogenic variants in genes such as *TSC1*, *TSC2*, *CDKL5*, *ARX*, *KCNQ2*, *STXBP1* and *SCN2A*. Understanding the genetic mechanisms of IESS may provide the opportunity to better discern IESS pathophysiology and improve treatments for this condition. This narrative review presents an overview of our current understanding of IESS genetics, with an emphasis on animal models of IESS pathogenesis, the spectrum of genetic aetiologies of IESS (i.e., chromosomal disorders, single-gene disorders, trinucleotide repeat disorders and mitochondrial disorders), as well as available genetic testing methods and their respective diagnostic yields. Future opportunities as they relate to precision medicine and epilepsy genetics in the treatment of IESS are also explored.

## 1. Introduction

The International League Against Epilepsy’s (ILAE) 2021 position statement on the classification of neonatal and infancy-onset epilepsy syndromes subdivides these conditions into two major groups: self-limited epilepsy syndromes and developmental and epileptic encephalopathies (DEEs) [1]. Infantile epileptic spasms syndrome (IESS) is classified as a DEE, emphasizing that affected infants experience impaired development related to both the epileptic encephalopathy and the underlying etiology independent of epileptiform changes. The classically known triad of West syndrome (WS) consists of epileptic spasms (seizures which are characterized semiologically by brief tonic axial contractions, typically occurring in clusters and often seen upon waking), as well as developmental regression or stagnation and the pattern of hypsarrhythmia on electroencephalogram (EEG) [2]. However, infants do not need to fulfil all WS criteria for diagnosis of IESS; epileptic spasms themselves may present prior to developmental concerns or hypsarrhythmia, and this flexibility in diagnosis recognizes the need for earlier treatment to optimize neurodevelopmental outcomes [3]. 

Onset of IESS typically occurs between 1 and 24 months of age, with the peak between 3 and 12 months [1]. IESS has an estimated incidence of 30 per 100,000 live births, and previous studies suggest a slightly higher incidence in males compared to females [4,5]. Many potential long-term sequelae have been observed in the IESS patient population, including lower intelligence quotient, increased rates of autism spectrum disorder, evolution to other epilepsy phenotypes and poor scores on quality-of-life metrics [6]. Previous studies have revealed that approximately 30% of patients may transition to a Lennox–Gastaut phenotype, a childhood-onset DEE characterized by multiple treatment-refractory combined generalized and focal seizure types, as well as cognitive impairment [1,7]. Moreover, IESS is also associated with increased rates of mortality; data from the United Kingdom Infantile Spasms Study revealed a mortality rate of less than 5% at 14 months old, and 8% by the age of 4 years [8,9].

An IESS phenotype can occur secondarily to a myriad of aetiologies. For instance, a recent cohort study of 541 IESS cases elucidated a definitive underlying cause in 53.2%, with the distribution as follows: 25.3% structural–acquired, 12.9% genetic, 7.2% genetic–structural, 5% structural–congenital, 2.4% metabolic, and 0.4% infectious [10]. Overall, based on previous studies, approximately 60% of cases of IESS are explained by structural, metabolic, or infectious causes, while the remaining 40% are secondary to underlying genetic mechanisms or remain undiagnosed [11]. Magnetic resonance imaging is considered first-line in the etiologic workup of IESS, as imaging is abnormal in one-half–two-thirds of children and can help to guide further investigations and treatment [1]. Research has revealed a meaningful diagnostic yield (approx. 25%) in IESS patients undergoing genetic testing via methods such as karyotyping, chromosomal microarray, single-gene testing, gene panels, whole-exome sequencing (WES) and whole-genome sequencing (WGS) [10,12]. In keeping with current ILAE guidelines, genetic investigations are considered in children for whom a definitive cause of IESS cannot be identified based on physical examination and imaging, or in those whose imaging findings may be characteristic of an underlying genetic condition [1]. The yield of genetic testing is highest in IESS when findings such as central hypotonia, dysmorphic features, autistic features, young age of spasm onset (i.e., <6 months) or developmental delay prior to onset of infantile epileptic spasms are present [13]. Current first-line treatments of IESS include steroids and vigabatrin; these modalities focus on the pathophysiologic mechanisms broadly implicated in the development of IESS, as opposed to targeting or correcting a specific underlying etiology [6]. Overall, response rates to current therapies vary significantly between studies and depending on the aetiology. For instance, previous research has shown adrenocorticotropic hormone (ACTH) to have an efficacy ranging from 36.7 to 87%, while vigabatrin response rates range between approximately 11 and 58% [6].

As evidenced above, ongoing advances in the field of neurogenetics are allowing a greater proportion of IESS patients to receive a definitive genetic diagnosis, which can have important implications with regard to prognostication and may also aid in our understanding of the pathophysiology of IESS. However, with the exception of a few key examples which will be discussed further, this work has not yet translated into an abundance of precision medicine techniques based on individual genetic diagnoses. In this review, we summarize the current understanding of the genetic mechanisms of IESS, the spectrum of genetic etiologies of IESS and currently available testing methods, as well as potential future directions in this field. Specifically, further advancements in our understanding of IESS pathophysiology and development of new treatment options are critical, particularly when considering the significant morbidity and mortality associated with an IESS phenotype.

## 2. The Growing Field of Epilepsy Genetics

Our level of understanding of the genetic underpinnings of epilepsy has completely transformed in the last two decades. Currently, over 900 monogenic epilepsy genes have been discovered and the majority (i.e., 90%) are associated with DEEs [14]. Over 70 pathogenic variants in epilepsy-related genes and over 28 copy number variants have been associated with IESS specifically, with many more potential candidate genes identified, and these numbers are expected to grow as our diagnostic tools continue to improve [12].

The pathophysiological mechanisms by which genetic changes lead to a DEE phenotype continues to be an active area of research; mechanisms of disease include, but are not limited to, channelopathies, disorders of synaptic function and disorders of protein translation, modification or transcription [15]. For instance, pathogenic variants in a number of voltage-gated sodium channel protein subunits, such as *SCN2A* and *SCN8A*, have been implicated in the development of DEEs and an IESS phenotype. As normal function of these channels is essential for action potential generation and propagation, it is not unexpected that channel dysfunction can lead to induction of irregular neuronal activity [16]. However, the exact mechanisms by which channelopathies result in the development of IESS is largely unknown [17]. Pathogenic variants in *DNM1* act as an illustration of impaired synaptic transmission resulting in the development of a DEE [18]. This gene leads to the expression of Dynamin-1, a pre-synaptic GTPase with a role in synaptic vesicle endocytosis and membrane recycling. Current research theorizes that Dynamin-1 dysfunction may lead to increased seizure risk through inefficient recycling and thus impaired tonic firing at inhibitory synapses. Pathogenic variants in *ARX*, a homeobox gene involved in the differentiation, migration and synaptogenesis of GABAergic interneurons via modulation of more broad-gene transcription, have also been implicated in the development of IESS and are further discussed [19]. The above monogenic causes of DEEs, and more specifically IESS, illustrate the concept that pathogenic variants in a wide range of genes, affecting many different aspects of neuronal function, can lead to a similar clinical phenotype; this concept is referred to as genetic heterogeneity [15]. However, it is important to highlight that, although genetic susceptibility to IESS exists, a second hit such as an additional environmental or genetic factor may be needed for the development of IESS to occur [17].

## 3. Mechanisms of IESS

While the pathogenesis of IESS is complex and incompletely understood at our current level of scientific understanding, multiple acute and chronic models exist which help to explain various disease aspects (Table 1). Alternatively, the multiple-hit and tetrodotoxin models can be described as lesional models, while the remaining described models are non-lesional in nature [17]. It is believed that multiple mechanisms derived from these models are important in the development of IESS, rather than one alone [17]. Ideal models of IESS include at least some of the following selection criteria: seizures which are specific to the epileptic stage, seizures which are spontaneous and spasm-like, interictal EEG resembling hypsarrhythmia as well as ictal EEG demonstrating an electrodecremental response, developmental regression or stagnation, and response to antiseizure medications used in the treatment IESS [20,21]. It is also important to note that manipulation of known IESS risk genes does not always generate useful animal models of spasms. For instance, *STXBP1* and *CDKL5* are two of the most commonly reported causes of IESS in humans; while genetic alterations in mice have led to various cognitive and morphologic changes, no spasms have been reported [22]. A succinct overview of some of the pathophysiological models of implicated in IESS is discussed herein.

### 3.1. Acute Models

Acute rodent models of IESS include the N-methyl-D-aspartate (NMDA) model, the NMDA model with prior prenatal betamethasone or perinatal stress exposure (known as the betamethasone/prenatal-stress NMDA model), and the γ-butyrolactone-induced IS model in the Down syndrome mouse [17]. These acute models manifest seizures in the weeks following induction [23].

#### 3.1.1. NMDA Models

In the NMDA model of IESS, administration of the excitatory glutamatergic agent in rodents induces emprosthotonic seizures which are semiologically similar to flexion tonic spasms (i.e., generally before postnatal age day 25) [17,24,25,26,27]. In mice models, NMDA administration has been observed to cause epileptic spasms in postnatal day 13 c57 mice, as well as deficits in motor and cognitive function [23]. Notably, administration of ACTH was observed to reduce the number of spasms in these mice [26]. Some limitations of this model include the fact that spasms do not develop spontaneously, and that the spasms are inconsistently responsive to ACTH [17]. Overall, these findings suggest that NMDA may have a role in the expression of spasms in IESS [23]. Furthermore, pathogenic variants in genes that encode subunits of the NMDA receptor, such as *GRIN2B* (encodes NR2B subunit), have been associated with the development of IESS in some cases [28].

In the betamethasone/prenatal-stress NMDA model, the accelerated onset and higher frequency of seizures in stress-related models supports the idea of a phenotypic interaction with perinatal stress [23]. Examples of stressors utilized in this model include the administration of betamethasone or forced restraint at gestational day 15 or forced exposure of pregnant mice to swimming in cold water [24,29,30,31]. Subsequently, similar to the aforementioned model, NMDA is administered postnatally to induce spasms. In this model, prenatal betamethasone brain priming, or restraint stress, has been shown to increase susceptibility to the development of NMDA-triggered spasms. Prenatal stress may alter neurotransmitter receptor expression as well as hormone levels in developing rats [30,32]. It was found that a single dose of ACTH was ineffective at managing spasms, while longer-term dosing of ACTH decreased the number of spasms as well as latency to spasm onset [30,32]. A limitation of this model is the lack of spontaneity of spasm development [17].

IESS occurs at a very active developmental stage, with important associated neurophysiologic changes including dendritic growth and myelination. Previous animal studies have shown that administration of ACTH enhances myelin turnover and dendritic sprouting, while supporting post-mortem studies of children with IESS have revealed impaired dendritic development within the cortex [33,34,35]. Moreover, decreased ACTH levels within the cerebrospinal fluid in patients with IESS, in conjunction with a rapid response to ACTH supplementation, provides evidence of the important role of the hypothalamic-pituitary axis in IESS pathogenesis [36]. A related molecular mechanism involves alterations in neurotrophic factors, endogenous molecules with a regulatory role in cell proliferation, differentiation and synaptogenesis [36]. Neurotrophic factors are important for the development of optimal neural connections, and abnormalities in various factor levels have been observed in IESS patients. For instance, neonates with hypoxic ischemic encephalopathy, a common etiology of IESS, have decreased brain-derived neurotrophic factor and β-nerve growth factor [37,38]. Previous studies have suggested various implications of neurotrophic factor dynamics in guiding the treatment of IESS (i.e., overproduction of β nerve growth factor could be a target for post-infectious and Tuberous Sclerosis Complex patients) as well as serving as a biomarker of disease severity (i.e., low cerebrospinal fluid insulin-like growth factor-1 levels have been associated with worse prognosis) [39,40,41,42].

#### 3.1.2. Down Syndrome GABA Model

There is a higher incidence of IESS in individuals with Down syndrome, a condition whose mouse model is known to have increased γ-aminobutryic acid (GABA) B-mediated potassium currents [23]. The Ts65Dn mouse model of Down syndrome has been studied as an animal model for the development of IESS and is sensitive to GABA_B_ receptor agonists. The G-protein-coupled inward-rectifying potassium channel subunit 2 (GIRK2) gene, *KCNJ6*, is also overexpressed in the model [43,44]. Administration of a GABA_B_ receptor agonist γ-butyrolactone in the Ts65Dn model, which is known to overexpress the GABA_B_ receptor and the GIRK2 subunit, induces extensor spasms and an electrodecremental response, similar to that of IESS, on EEG. However, mice that are GIRK2 (−/−) have been found to be resistant to GABA_B_ agonist development of spasms and EEG changes. Therefore, the presence of the GIRK2 channel is required for the development of IESS in the GABA_B_ receptor agonist Ts65Dn mouse model of Down syndrome, a finding which may have therapeutic importance in the treatment of IESS [43]. Some limitations of this model include the fact that spasms are not spontaneous, that a hypsarrhythmia background rhythm is not present on EEG, and that long-term epilepsy or behavioural changes have not been reported [17].

The role of the GABA_A_ receptor in spasm development has also been studied; this receptor initially functions in an excitatory manner in the neonatal period and infancy until a “GABA_A_ switch” occurs, at which point in time its functionality becomes inhibitory in nature [45]. On a microscopic level, this channel initially facilitates outflow of chloride ions, thus leading to cell depolarization and excitation; with the GABA_A_ switch, an alteration in chloride transporter isoform expression allows this channel to assume its hyperpolarizing, inhibitory role mediated by chloride ion influx [45]. With regard to IESS development, previous studies have shown alterations in neurosteroid sensitivity and decreased expression of GABA_A_ receptors in IESS models and from a management perspective, one effective treatment of spasms is the GABA transaminase inhibitor, vigabatrin [36,46,47]. Interestingly, pathogenic variants in a number of GABA-related epilepsy genes have been implicated in the development of IESS, such as pathogenic variants in *GABRB1*, *GABRA1* and *GABRB3*, highlighting the importance of GABA-related transmission in the development of IESS [48,49,50,51].

### 3.2. Chronic Models

Several chronic models have also been studied in the pathophysiology of IESS. Chronic models include the tetrodotoxin rat model, *ARX* mouse model, and multiple-hit rat model of IESS [23]. In the tetrodotoxin model, chronic infusion of the sodium channel blocker tetrodotoxin in infant rodent models has been observed to result in the development of spasms at a minimum of 10 days after infusion, and EEG features of hypsarrhythmia [52]. Specifically, this model has been useful in monitoring the ictal and interictal EEG patterns seen in IESS in older animals, particularly considering that a lack of hypsarrhythmia development is a significant limitation of other animal models [17,23]. The tetrodotoxin model has also been reported as being responsive to vigabatrin treatment [53]. A limitation of this model is the late onset of spasms [17].

Pathogenic variants in *ARX*, a homeobox gene involved in the differentiation, migration and synaptogenesis of GABAergic interneurons, via modulation of more broad-gene transcription, have also been implicated in the development of IESS [19]. The *ARX* gene encodes a transcriptional factor involved in GABAergic progenitor migration and cholinergic neuron differentiation, and the *ARX* mouse model allows us to better understand the association between interneuronopathy and spasm development [23,54]. The *ARX* knock-in model (i.e., trinucleotide repeat expansion) as well as the knockout model (i.e., deletion from the ganglionic eminence) both show evidence of interneuronopathy and display an IESS phenotype clinically. In comparison, another mouse model without interneuronopathy does not develop spasms [55,56,57]. A limitation of both the knock-in and knockout models is a lack of sufficient data to allow for the extrapolation of research to other genetic aetiologies; moreover, the knockout model also has a late onset of spasms and paucity of long-term behavioural changes noted [17].

Finally, the multiple hit model in rats is a chronic model used to exemplify refractory IESS secondary to structural lesions [23]. This protocol involves an intracerebroventricular infusion of doxorubicin and intracortical infusion of lipopolysaccharide on postnatal day 3 to target the grey and white matter, respectively. On postnatal day 5, a tryptophan hydroxylase inhibitor injection is given to deplete serotonin, since metabolism of this neurotransmitter is abnormal in infants with IESS [58,59]. The evolving phenotype generated by this model includes spasms from postnatal days four through 13, multiple other seizure types starting on postnatal day 9 and adult epilepsy [23]. A limitation of this model is that hypsarrhythmia has not been conclusively identified in previous studies [17]. Preclinically, this model has been used to screen for the utility of various pharmacologic agents in treating IESS [60,61].

Overall, the acute and chronic models exemplify the intricate, multifactorial landscape of IESS pathogenesis. Broadly, a recent comparative analysis of infants with IESS versus those with other seizure types revealed that IESS has a preferential association with overarching developmental and regulatory pathways, cell cycle regulatory and tumerogenic pathways and general immunologic processes [62]. It has been proposed that this finding provides mechanistic evidence for the developmental desynchronization hypothesis of IESS development, which implies that IESS may result from a temporal disconnect in multiple critical neurodevelopmental processes, thereby leading to disturbances in brain function [63].

## 4. Spectrum of Genetic Aetiologies of IESS

Even within the subcategory of genetically based IESS, multiple different aetiologies exist; these include chromosomal disorders, single-gene variants, trinucleotide repeat disorders and mitochondrial disorders (Table 2). While it is beyond the scope of this review to describe all aetiologies of IS in detail, prototypical examples within each category have been provided.

### 4.1. Chromosomal Disorders

Individuals with Down syndrome (i.e., Trisomy 21) are at significantly increased risk of epilepsy compared with the general population; IESS is the most frequently encountered epilepsy syndrome in individuals within this patient group, with an estimated incidence of 0.6–13% [64]. As discussed previously, one animal model of Down syndrome focusses on the proposed role of GABAergic signalling in spasm pathogenesis [23]. A literature review conducted by Kats et al. revealed an 81% response rate to ACTH in this population, as well as better outcomes in terms of relapse rates and intractable epilepsy development compared to those with IESS of unknown etiology [65]. However, a recent 10-year retrospective review found that, at follow up of a median of approximately 2 years of age, 25% of their cohort had ongoing seizures and 85% had developmental concerns [66]. A single case report has documented an association between IESS and another aneuploidy, Klinefelter syndrome (47, XXY karyotype), which was responsive to ACTH therapy [67]. IESS has also been described in infants with Trisomy 18 [68].

To date, over 28 copy number variants (CNVs), structural variants which are detectable by both chromosomal microarray and WGS methods, have been associated with an IESS phenotype [12]. While not exhaustive, a sample of CNVs which have been reported in multiple cases of IESS include 1p36 deletion, 7q11 deletion, 17p13.3 deletion (Miller–Dieker syndrome), 2q24.3 duplication, 15q11.2 duplication, Xq27.2q28 duplication and tetrasomy 12p (Pallister–Killian syndrome) [12,19]. With the exception of Miller–Dieker syndrome, in which spasms are thought to be related to impaired glutamatergic and GABAergic interneuron migration in the context of *PAFAH1B1/LIS1* deletion, the biological mechanisms of IESS in this population are incompletely understood [69]. Largely, research suggests IESS development in these patients is related to changes in key genes (for instance, an Xp22.13 deletion reported by Peng et al. included exon 1 of *CDKL5*, which is strongly associated with IESS) within a relevant developmental context, and differences in phenotypes between individuals with the same diagnosis may be mediated by “multiple hit” genomic changes or other factors (i.e., environmental, epigenetic) which influence the development of IESS [10,12].

### 4.2. Single-Gene Disorders

An increasing number of single-gene disorders are being recognized as being associated with an IESS phenotype [10,12,13,70,71,72,73,74,75]. Discussion of all attributable genes is beyond the scope of this review; we will focus on a sample of those which have been most commonly reported in the literature. Overall, some of the genes most frequently associated with IESS include pathogenic variants in *TSC1*, *TSC2*, *CDKL5*, *ARX*, *KCNQ2*, *STXBP1*, *SCN2A* and *SCN8A*, amongst others, although exact incidences of variants fluctuate between individual cohort studies [10,12,13,70,71,72,73,74]. Moreover, genes associated with IESS have various patterns of inheritance, including de novo or autosomal dominant, autosomal recessive and X-linked patterns [10,12,13,70,71,72,73,74].

Previous research has elucidated that individuals with TSC, which occurs as a de novo pathogenic variant or is inherited in an autosomal dominant manner, have around a 50% risk of developing IESS [76]. From a pathophysiologic viewpoint, this condition represents an etiology with spasm development secondary to a structural–genetic mechanism. In TSC, excessive activity within the mammalian target of rapamycin (mTOR) pathway secondary to a loss of function variant in either *TSC1* or *TSC2* leads to abnormal cell growth, differentiation, and migration; ultimately, this precipitates development of lesions such as tubers, subependymal nodules and radial migration lines [76]. The location of tubers is quite heterogeneous, and a recent network analysis study showed that, while no single location was associated with the development of IESS, over 95% of tubers in patients with spasms had functional connectivity with the globi pallidi and cerebellar vermis [76]. Another mechanism involved in IESS pathophysiology in this patient population is abnormal GABAergic neurotransmission; previous studies have shown dysregulation of interneuron generation as well as a delay in the maturation of GABAergic networks [77]. Vigabatrin, a GABA–transaminase inhibitor, is known to be particularly efficacious in TSC patients; in addition to elevating brain GABA levels by inhibiting enzymatic degradation of this substrate, it also inhibits mTOR pathway activity specifically [77].

Examples of other genes associated with IESS, which are mechanistically involved in GABAergic transmission, include *GABRB1*, *GABRB3* and *GABRA1*, amongst others [48,49,50,51]. Specifically, the aforementioned genes encode subunits of GABA receptors. Pathogenic variants within GABA subunits leads to downregulation of receptor expression and subsequent reduction in the amplitude of GABA-evoked potentials; this net loss of inhibitory signalling facilitates the development of epilepsy [78]. Outside of the context of IESS, case reports of a patient with Lennox–Gastaut syndrome secondary to a pathogenic *GABRB3* variant, as well as another patient with *GABRA1*-related drug-resistant focal epilepsy, revealed a significant positive effect with use of vinpocetine supplementation [79,80]. This alkaloid compound derived from the periwinkle plant was trialled as it had been shown to enhance GABA channel conductance in earlier cell models. Moreover, specific truncation variants of *GABRB3* with clear loss of function (LOF) respond well to treatment with vigabatrin, while treatment with vigabatrin in the case of gain of function (GOF) variants has led to hypersensitivity with resultant hypotonia, sedation and respiratory depression in some patients [81]. This is thought to be due to further exacerbation of GABAergic tonic currents in patients with GOF variants, and again highlights how understanding genotype can influence patient care. Finally, variants within genes involved in the regulation of excitatory neurotransmission have also been implicated in the development of IESS; these include *CACNA1A*, *KCNB1*, *SCN2A*, *SCN8A* and *KCNQ2*, amongst others [10].

*STXBP1* is an example of gene involved in presynaptic processes, which is increasingly being recognized one of the most common causes of genetic IESS based on multiple cohort studies [10,12,13]. This gene encodes the protein MUNC18-1, which plays an integral role in cellular transport, exocytosis, and neuronal viability [82]. *STXBP1* haploinsufficiency is associated with an inhibition-dominated network pattern on EEG; supportive evidence from molecular studies has revealed downregulation of glutamatergic synapses in conjunction with upregulation of GABAergic synapses in a human cell assay [83,84]. Overall, these changes are thought to predispose to dyssynchronization in developing neuronal circuits, thus causing an increased risk of seizures and IESS [85]. De novo pathogenic variants within *STXBP-1* have a broad clinical phenotype, including features such as intellectual disability, movement disorders, gross motor dysfunction, early-onset DEEs, and difficulties with communication [85]. Personalized treatment strategies under investigation include the possibility of chaperone proteins to help stabilize abnormal mutant protein caused by missense variants in *STXBP1* [86].

*CDKL5* is another example of an IESS-associated gene involved in multiple cellular processes, including aspects of neuronal development such as migration, axonal and dendritic formation and synaptogenesis, as well as later synaptic function [87]. *CDKL5* deficiency disorder is inherited in an X-linked manner and was previously considered to be a variant of Rett syndrome. Within this condition, seizures typically start very young (i.e., median age of onset 6 weeks), and its associated epilepsy phenotype has been described as progressing through three stages: early-onset convulsive seizures, then epileptic spasms and finally ongoing treatment-refractory seizures thereafter [88]. Ganaxolone, a neuroactive steroid which enhances GABA inhibition by positive allosteric modulation of both extrasynaptic and synaptic GABA_A_ receptors, may represent a possible targeted treatment option for *CDKL5*-related DEEs and will be further discussed later in this review [89].

### 4.3. Trinucleotide Repeat Disorders

Trinucleotide repeat disorders can cause pathology via multiple mechanisms including LOF due to transcription repression, GOF due to sequestration of RNA-binding proteins or translation of proteins harbouring repeats and toxicity of translated repeat peptides themselves [90]. As discussed previously, *ARX* is an X-linked gene implicated in multiple aspects of brain development, most notably related to GABAergic function [54]. Structurally, the *ARX* gene itself is noted to include five exons containing four polyalanine tracts. In addition to evidence of pathogenic single nucleotide changes resulting in missense or nonsense variants at various regions within the gene, and leading to a wide range of clinical phenotypes, IESS has also been observed in patients with expansion of the GCG trinucleotide repeat within the first polyalanine tract of *ARX* [91]. One study revealed that, in six boys harbouring this expansion, there was a consistent phenotype of severe global developmental delay, IESS and severe dyskinetic quadriparesis; moreover, 50% of the sample developed recurrent status dystonicus [91].

IESS has also been associated with massive CAG expansions in *ATXN2*, the gene associated with autosomal dominant spinocerebellar ataxia type 2 [92]. In individuals with 33–64 repeats, this disease typically manifests in adulthood with slowly progressive oculomotor abnormalities, ataxia and dysarthria. A cohort study of six patients with more than 200 CAG repeats revealed a severe phenotype of encephalopathy with dysautonomia, as well as retinitis pigmentosa in 66% and IESS in 50% [92]. Bioinformatics analysis was completed, which revealed that the severe phenotype observed may be due to impaired postsynaptic vesicle endocytosis via disruption of *ATXN2*′s interactions with postsynaptic structural proteins *SPTAN1* and *MAGI2* [92]. Additionally, pathogenic variants in *SPTAN1* are associated with a constellation of phenotypic and neuroimaging findings including an IESS phenotype, as well as acquired microcephaly, intellectual disability, hypomyelination and cerebellar atrophy [93,94].

### 4.4. Mitochondrial Disorders

IESS secondary to mitochondrial disorders is less commonly reported. For instance, in cohorts of 124 genetically confirmed IESS patients and 541 IESS patients of diverse aetiologies, only 1 patient from each group was reported as having an underlying mitochondrial disorder [10,13]. Specifically, variants in *MT-ND5* and *MT-ND1* were identified in the respective studies. Multiple previous cases of IESS in the context of Leigh syndrome have been reported in the literature [95,96]. Leigh syndrome is a neurodegenerative mitochondrial disorder characterized by symmetric basal ganglia lesions and a spectrum of clinical features including hypotonia, psychomotor regression and feeding concerns [95]. Pathophysiologically, Leigh syndrome is caused by failure of energy generation processes on a cellular level; it can be due to deficiencies of pyruvate dehydrogenase, mitochondrial DNA defects or issues with assembly of respiratory chain complexes, amongst other mechanisms [97]. In one study comparing Leigh syndrome patients with and without associated IESS, those with IESS had earlier onset Leigh syndrome, but there was ultimately no effect of spasm development on prognosis [96].

### 4.5. Candidate Genes

It is of note that many more candidate genes continue to be identified in cohort studies. For instance, in a recent cohort of only 21 IESS patients undergoing WES, five candidate variants were detected in the genes *PEMT*, *DYNC1I1*, *ASXL1*, *RALGAPB* and *STRADA* [70]. With ongoing use of genetic technologies in the workup of IESS, increasing evidence will become available to allow for more definitive genetic diagnosis in these uncertain cases. While there is a wide array of pathophysiologic mechanisms of IESS, it is possible that a higher proportion of candidate genes will be found in pathways affecting broader developmental processes and cell body organelles such as the Golgi apparatus and endoplasmic reticulum, as these were found to be more strongly associated with IESS development in a study conducted by Berg et al. [62]. Moreover, this study also demonstrated that individuals with IESS had enrichment of regulatory targets of microRNAs involved in various cell functions such as growth, proliferation and response to oxidative stress. Overall, these findings suggest a role of epigenetics in the development and pathophysiology of IESS. Targeted microRNA therapy is being investigated as a potential future treatment option for genetic epilepsies and could be utilized in IESS in the future. For instance, this strategy could be used to selectively upregulate transcription of a specific gene in the context of a disorder of haploinsufficiency [98].

## 5. Genetic Testing for IESS

Families experience a significant diagnostic odyssey when navigating the process of neurogenetic testing, and there are many important considerations which must be addressed. The importance of pre- and post-testing genetic counselling cannot be underestimated; this allows families to gain an understanding of indications for testing, potential findings (i.e., positive result, negative result, variant of uncertain significance), the implications of incidental findings (e.g., carrier status for an unrelated recessive condition), potential impacts on family planning and the current status of precision medicine in genetic epilepsies [99]. Importantly, a cohort study of 124 genetic IESS cases revealed a substantial time period between spasm onset and genetic diagnosis, with median age of seizure onset at 5 months old versus time of confirmed etiologic diagnosis at 12 months of age [13]. In the era of precision medicine, identification of an underlying genetic etiology may have treatment and prognostic implications. Genetic testing should be considered in children for whom a definitive cause of IESS cannot be identified based on physical examination and imaging, or in those whose imaging findings may be characteristic of an underlying genetic condition [1].

Multiple genetic testing methods are currently available for the etiologic investigation of IESS. The yield of individual testing methods shows some variability between cohort studies (Table 3). As an example, one study found the following yields: 1.1% for karyotyping, 5.8% for chromosomal microarray, 25.7% for epilepsy panel, 26.9% for whole-exome sequencing and 2.9% for mitochondrial genome analysis [10]. Karyotyping, the process of pairing and ordering chromosomes, is used to assess for major chromosomal abnormalities such as aneuploidies, structural rearrangements, or deletions or duplications greater than 5 Mb [100]. In the context of characteristic facial dysmorphisms and systemic examination findings consistent with a diagnosis of Down syndrome, yield of this investigation is generally very high [12]. However, outside of the setting of Down syndrome, the yield is closer to 1% in IESS [10].

IESS has also been associated with an array of CNVs, such as microdeletions or microduplications, which can be detected via chromosomal microarray. In a cohort study of 728 IESS patients (wherein 587 had genetic testing), 13 (2.2%) ultimately received a genetic diagnosis of a CNV; in another study, a diagnostic yield of 5.8% was found for chromosomal microarray in patients who had remained undiagnosed following first-tier investigations such as MRI [10,12]. While chromosomal disorders and CNVs account for a smaller proportion of IESS cases, karyotyping and chromosomal microarray are readily available, cost-effective and relatively timely testing modalities, and should be considered in the appropriate clinical context. Single gene sequencing can also have a high diagnostic yield in light of specific clinical features; for instance, Liu et al. found a diagnostic yield of 83.5% for IESS patients with a clinical diagnosis of TSC (n = 127) undergoing *TSC1* and *TSC2* variant testing, as well as a yield of 100% (n = 2) for individuals with multiple café-au-lait spots undergoing *NF1* variant testing [12].

Beyond this initial stage of genetic testing methods, investigations for still-unsolved cases of IESS include gene panels, WES and WGS. While the exact diagnostic yield for these modalities varies slightly between cohort studies to date, the significant insight which advanced genetic techniques provide into previous undiagnosed cases of IESS cannot be argued. For instance, Peng et al. revealed a conclusive genetic diagnosis in 25.7% of 305 patients with IESS undergoing customized multigene epilepsy panels, and 26.9% through WES [10]. In a smaller cohort of 21 IESS patients, pathogenic or likely pathogenic variants, and thus established diagnoses, were found in 5 patients (23.8%) via analysis of WES; another 5 patients (23.8%) were found to have variants of uncertain significance felt to be candidate genes for IESS [70]. WES is able to assess for variants in coding regions of the genome; in contrast, benefits to WGS include increased consistency of coding region coverage, improved detection of structural variants such as CNVs, and identification of disease-causing variants within non-coding regions (although clinical interpretation of non-coding variants remains challenging) [101,102].

**Table 3:** Examples of diagnostic yields of genetic testing modalities in IESS.

Study	Karyotype	CMA	Epilepsy Panel	WES	WGS	Mitochondrial Genome Analysis
Peng et al. (2022) [10]	*n* = 2/183 yield = 1.1%	*n* = 12/207 yield = 5.8%	*n* = 27/105 yield = 25.7%	*n* = 63/234 yield = 26.9%		*n* = 1/34 yield = 2.9%
Lee et al. (2022) [103]					*n* = 4/16 yield = 25% n = 6/16 if including candidate genes yield = 37.5%	
Liu et al. (2021) [12]			*n* = 24/289 yield = 8.3%			
D’Gama et al. (2023) [104]					*n* = 6/32 yield = 19%	
DeMarest et al. (2021) [70]				*n* = 5/21 yield = 23.8% *n* = 10/21 if including candidate genes yield = 47.6%		

Legend: CMA = chromosomal microarray; WES = whole-exome sequencing; WGS = whole-genome sequencing.

To date, there is limited research on the utility of WGS in IESS specifically; one study utilizing a whole-genomic approach in a cohort of 16 IESS patients had a diagnostic yield of 25%, while another had a yield of 19% [103,104]. These results are similar to those of previous WES studies. However, researchers in the first study also identified two candidate variants of uncertain significance in *SHROOM4* and *SOX5*, which were both located within intronic regions. Another study by Palmer et al. examined the utility of WGS after negative WES in DEEs broadly; of these 15 undiagnosed patients, 3 individuals (20%) were found to have complex structural variants which would not have been detected by WES [71].

Another important issue to consider is the cost-effectiveness of various diagnostic methods. While not examined in IESS specifically, studies to date have focussed mostly on the use of CMA, gene panels and WES in epilepsy in general [105,106,107,108,109]. Research suggests that early utilization of WES or large-phenotype-driven panels are more cost-effective first-tier investigations, which is not necessarily in line with current guidelines. Less is known about the cost-effectiveness of WGS; one study comparing the costs associated with WGS versus WES plus CMA in the context of workup for autism spectrum disorder found no significant difference between the two strategies [110].

## 6. The Future of Genetics in IESS

### 6.1. Opportunities for Precision Medicine

Current first-line treatments of IESS include ACTH, prednisolone, and vigabatrin [6]. Based on prospective studies to date, the response rates for these treatments are as follows: 36.7 to 87% for ACTH, 11 to 70% for prednisolone, and 11 to 58% for vigabatrin [6,111,112,113,114,115,116]. The first two therapies focus on broadly targeting the immune-mediated pathogenesis of IESS, while vigabatrin targets the GABAergic network involved in the development of spasms. Based on multiple studies, vigabatrin is noted to be particularly efficacious in the TSC patient population and is considered the first choice of treatment in this group [77]. Importantly, current pharmacologic treatment modalities have a high rate of relapse after initial response; for instance, various studies assessing ACTH treatment found relapse rates ranging from 15 to 34% [6]. There is also a high side effect burden associated with these medications; major side effects of ACTH include immunosuppression, hypertension, adrenal insufficiency, cardiomyopathy, gastrointestinal problems and electrolyte imbalances, while vigabatrin can lead to irreversible peripheral visual field defects and structural changes on brain MRI consistent with intramyelinic edema [6]. Additional potential treatment modalities include surgical resection for those with lesion-related epileptic spasms, initiation of the ketogenic diet, as well as other antiseizure medications including nitrazepam, levetiracetam, valproic acid, topiramate, clobazam, rufinamide, zonisamide and perampanel in the case of refractory IESS [6,111]. One recent study examined outcomes after surgical treatment of lesional IESS in a cohort of 19 patients; 79% of patients achieved seizure freedom after surgical intervention, while 74% also showed developmental improvement [117]. Despite advancements in our knowledge of the causes of IESS, this has not yet translated into an abundance of novel therapeutic options. Specchio et al. argue that this lag in precision therapies may be due to the significant variability in terms of aetiologies, as well as a paucity of reliable animal models, most of which only exhibit some features of IESS pathogenesis [118].

TSC is an example of an etiology which, based on our understanding of underlying disease-causing mechanisms, has entered an era of more targeted treatments; examples include pre-emptive therapy with vigabatrin as well as initiation of mTOR inhibitors [119]. As discussed previously, in addition to its effects on GABAergic transmission, vigabatrin has also been shown to have inhibitory effects on the mTOR pathway specifically [77]. The utility of pre-emptive vigabatrin treatment at onset of epileptiform activity on EEG has been investigated through the recent EPISTOP and PREVeNT trials [120,121]. The EPISTOP randomized control trial revealed a delayed time to first clinical seizure with vigabatrin prophylaxis, as well as reduced risk of clinical seizures, drug-resistant epilepsy and IESS at 24 months-old [121]. However, not all of these findings were reinforced by the PREVeNT trial; while a delayed onset and decreased incidence of IESS were supported with preventative vigabatrin, there were no alterations in incidence of focal seizures, drug-resistant epilepsy, or neurocognitive outcomes at 24 months of age [120]. With research regarding outcomes with early intervention ongoing, many clinicians perform EEGs in asymptomatic individuals to monitor for development of hypsarrhythmia in the context of genetically confirmed TSC; as such, earlier genetic diagnosis can have an impact on time of treatment. With regard to mTOR inhibitors, everolimus and sirolimus are two agents with Food and Drug Administration approval to treat various systemic manifestations of TSC, including subependymal giant cell astrocytomas, angiomyolipomas and lymphangioleiomyomatosis [119]. Studies have examined the effectiveness of these agents in treating TSC-related epilepsy, and they are currently considered as adjunctive therapy in those with drug-resistant epilepsy [122,123,124]. Moreover, clinical trials are also in process to examine the effect of mTOR inhibitors such as sirolimus on preventing and delaying the onset of seizures and possibly IESS in infants with TSC (Clinical Trial Gov, NCT05104983).

With regard to other currently utilized precision medicine strategies, some examples exist. Pyridoxine and pyridoxyl-phosphate supplementation are examples of treatments used in the context of *ALDH7A1* and *PNPO* pathogenic variants, respectively, and lead to resolution of IESS by countering the specific metabolic pathway defects involved [125]. For individuals with DEEs related to GOF variants in *SCN2A* and *SCN8A*, sodium channel blockers may be beneficial in the management of epilepsy; in contrast, with LOF variants in *SCN2A* and *SCN8A*, sodium channel blockers may worsen the epilepsy phenotype [126,127]. Patients with GOF variants in *SCN2A* (i.e., onset before three months) and *SCN8A* (i.e., onset in the first year) present early with infantile epilepsy with variable severities, while those with LOF variants present later in childhood. IESS, along with other DEEs, have been described in individuals harbouring pathogenic *SCN2A* and *SCN8A* variants [126,127]. Although sodium channel blockers may be beneficial in the treatment of epilepsy related to GOF variants in *SCN2A* and *SCN8A*, it is also known that sodium channel blockade may induce spasms or worsen them [128].

As another example of strategic therapy, within animal models, neonatal estradiol has been shown to reverse GABAergic neuronal decline in the context of *ARX* trinucleotide repeat expansion specifically [129]. Given the underlying pathophysiology of IESS in Down syndrome, GABA_B_ receptor antagonists could also be useful in the management of IESS related to Down syndrome [17]. Targeting the GIRK2 channel via antagonism may also be a potential avenue of treatment as this channel is required for the development of spasms in the GABA_B_ receptor agonist-induced mouse model [43]. Furthermore, ganaxolone (3α-hydroxy-3ß-methyl-5α-pregnan-20-one), a neuroactive steroid which enhances GABA inhibition by positive allosteric modulation of both extrasynaptic and synaptic GABA_A_ receptors, may also be utilized in the treatment of IESS. Recently, ganaxolone has been shown to result in a significant decrease in major motor seizure frequency in children with *CDKL5* deficiency disorder [89]. Given the high rates of IESS in individuals with *CDKL5* disorder, it is possible that future studies may investigate the use of ganaxolone in its specific treatment. A previous small study of twenty children over a decade ago demonstrated some efficacy of ganaxolone in reducing infantile epileptic spasms in those with refractory IESS. However, additional studies are needed to replicate these findings [130].

As previously described, the use of gene ontology and pathway enrichment analysis has also been used to compare cellular function between spasm and non-spasm epilepsy patients and found that there were differences in terms of pathways and cell regions or organelles affected between these two groups [62]. Specifically, IESS was associated with broader developmental pathways, as well as neuronal cell body organelles including the Golgi apparatus and endoplasmic reticulum [62]. It has been proposed that understanding these differences in biologic underpinnings is key to advancing the development of precision medicine in IESS by allowing for more targeted treatment of underlying pathology on a molecular basis [62]. Individuals with IESS have also been found to have enrichment of regulatory targets of microRNAs involved in various cell functions such as growth, proliferation, and response to oxidative stress. Targeted microRNA therapy is being investigated as a potential future treatment option for genetic epilepsy and could be utilized in IESS in the future [62]. For instance, this strategy could be used to selectively upregulate transcription of a specific gene in the context of a disorder of haploinsufficiency [98]. Currently, there are no commercially available miRNA targeted therapies, but multiple preclinical through Phase II clinical trials are ongoing for diseases including hepatitis C, various cancers and amyotrophic lateral sclerosis [131].

Finally, a future direction of targeted personalized IESS treatment involves the development of gene therapy to treat monogenic conditions. Adenovirus-associated virus (AAV) gene replacement has been approved for and completely redefined clinical outcomes for spinal muscular atrophy and is being studied for genetic epilepsies such as Dravet syndrome [132,133]. AAV gene replacement therapy can be used for the delivery of genes encoding neutrophic factors, enzymes, potassium channels, and neuromodulatory peptides, as examples [134]. A recent review discussing the genetics of *SLC13A5* deficiency disorder, a DEE caused by deficiency of the sodium/citrate cotransporter, highlighted some logistical considerations for the development of AAV-based gene therapy which can be applied when considering gene therapy in the IESS population [135]. First, due to the size of AAV vectors, genes must be less than 5 kilobases in order to be packaged. Preclinically, the availability of accurate animal models is crucial to understand treatment efficacy [135]. There are also important safety considerations, including the use of pharmacology and toxicology studies to assess optimal dose for humans, as well as optimal age of delivery in order to maximize benefit while minimizing potential adverse effects. In addition to gene therapy, a number of cell-based therapies are also being studied in the treatment of epilepsy and could be studied in the IESS population in the future. Cell- based therapies include a number of approaches such as the transplantation of mesenchymal stem cells, neural stem cells and bone marrow mononuclear cells, as examples [134]. Encapsulated cell biodelivery, which involves the implantation of a capsule with a semipermeable membrane containing genetically modified cells that secrete therapeutic substances, is also being studied as a potential epilepsy therapy [134].

### 6.2. Opportunities for Gene Discovery

Despite ongoing advancements in genetic diagnostic tools, as detailed in this review, a significant proportion of presumed genetically based IESS continue to remain undiagnosed. For example, in a cohort of 541 patients, 46.7% remained undiagnosed following workup including basic laboratory investigations, metabolic screening, neuroimaging and genetic testing [10]. Current and future strategies to improve diagnostic yield in DEEs, as highlighted by Johannesen et al., include reanalysis of older WES or WGS, investigation for somatic mosaicism, completion of long-read sequencing to assess for tandem repeats or other structural variants which can be missed by WES or WGS, and consideration of complementary genetic tests such as analysis of the transcriptome, metabolome and DNA methylation signatures [136]. The aforementioned complementary genetic testing methods have not yet been investigated extensively in the IESS population.

The standards of diagnostic workup are subject to change with the advancement and availability of diagnostic tools; for instance, Lee et al. utilized a whole-genomic approach in diagnosing IESS and found pathogenic or likely pathogenic variants in 25% of their sample (four patients), as well as two additional intronic candidate variants [103]. They argue that, given the significant genetic heterogeneity of this patient population, further genome-based studies are necessary to improve diagnostic rates as well as to assist in improving clinical interpretation of variants within non-coding regions. However, it is important to highlight that challenges exist with regard to access to diagnostic testing, such as genetic and metabolic testing, in many parts of the world for clinicians caring for children with IESS. Given these challenges, global efforts are needed to increase the affordability, access, and resources necessary to interpret the results of genetic testing in the setting of DEEs like IESS [137].

## 7. Conclusions

In summary, IESS is a DEE with significant neurodevelopmental implications [1,6]. There are various causes of IESS, but approximately 40% of cases are of genetic or currently of unknown etiology [11]. With the continued advancement of genetic diagnostic technologies, the proportion of previously unknown IESS aetiologies receiving confirmatory genetic diagnoses continues to grow. In this review, we have highlighted some of the currently available genetic testing methods, as well as the spectrum of underlying genetic changes resulting in an IESS phenotype [10]. Current pharmacologic standards of treatment for IESS include ACTH, prednisolone, and vigabatrin, which broadly target pathophysiologic mechanisms attributed to the development of IESS including abnormalities within immune-mediated and GABAergic regulation [6]. Moreover, these therapies are associated with high rates of IESS relapse as well as a significant side effect profile. Precision-based therapies for IESS are currently lacking and developmental prognosis is often guarded. With ongoing advancements in genetic testing techniques, we hope that this will translate into the development of more targeted precision medicine therapies in the foreseeable future [136,138]. Examples of currently utilized personalized medicine techniques based on genetic information include, as examples, the use of mTOR inhibitors in patients with TSC or supplementation of pyridoxine in the context of *ALDH7A1* pathogenic variants [122,125]. Ultimately, the development of gene therapy for monogenic causes of IESS or cell-based therapies are promising prospects and require further research efforts [135].

## Figures and Tables

**Table 1:** Examples of animal models of IESS.

	Animal Model	Protocol	Clinical Importance
Acute Models	N-methyl-D-aspartate (NMDA) model	Administration of NMDA postnatally induces emprosthotonic seizures (similar to flexion tonic spasms)	Suggests role of NMDA in spasm expression
	Betamethasone/prenatal-stress NMDA model	In addition to postnatal NMDA administration, mice exposed to prenatal stressors such as betamethasone, forced restraint and forced swimming in cold water	Faster-onset and higher-frequency seizures suggest phenotypic interaction of spasms with perinatal stress
	Ts65Dn mouse model of Down syndrome	Administration of GABA_B_ receptor agonist induces extensor spasms and electrodecremental response on EEG; effect not seen in mice that are GIRK2 (−/−)	Provides evidence that GIRK2 channel subunit is required for development of spasms in this model
Chronic Models	Tetrodotoxin rat model	Chronic infusion of sodium channel blocker results in development of epileptic spasms and hypsarrhythmia	Useful in monitoring ictal and interictal patterns seen in IESS in older animals
	*ARX* mouse model	Mutant mice with non-functional *ARX* gene, which typically has role in GABAergic differentiation and migration	Provides evidence for association between interneuronopathy and spasm development
	Multiple-hit rat model	Postnatal intracranial infusion of doxorubicin and lipopolysaccharide to target grey and white matter, respectively, followed by injection of tryptophan hydroxylase inhibitor to deplete serotonin	Model of refractory IESS secondary to structural lesions

**Table 2:** Examples of some of the reported genetic IESS aetiologies.

Chromosomal disorders		Trisomy 18; Trisomy 21; 47, XXY (Klinefelter syndrome); 2q24.3 duplication; 5p12-11 duplication; 15q11.2 duplication; Xq27.2q28 duplication; 1p36 deletion; 3p25.3 deletion; 4q32.3q35.1 deletion; 7q11 deletion; 9q33.3-34.11 deletion; 9p24.3-22.3 deletion; 17p13.3 deletion (Miller–Dieker syndrome); 20q13.3 deletion; Xp22.13 deletion; tetrasomy 12p (Pallister–Killian syndrome)
Single-gene disorders	De novo, dominant	*ALG13*; *CACNA1A*; *CHD2*; *CDK19*; *CSNK2A1*; *CYFIP2*; *DNM1*; *EHMT1*; *FOXG1*; *GABBR2*; *GNAO1*; *GNB1*; *GRIN1*; *GRIN2B*; *HDAC4*; *IRF2BPL*; *KANSL1*; *KCNB1*; *KCNMA1*; *KCNT1*; *KCNQ2*; *KMT2C*; *KMT2D*; *KMT2E*; *MBD5*; *MEF2C*; *NEDD4L*; *NF1*; *NPRL3*; *NTRK2*; *PACS2*; *PPP2R1A*; *PPP3CA*; *PRRT2*; *PURA*; *SATB1*; *SCN1A*; *SCN2A*; *SCN8A*; *SLC2A1*; *SLC35A2*; *SMARCA2*; *SPTAN1*; *STXBP1*; *SYNGAP1*; *TCF4*; *TSC1*; *TSC2*; *TUBB2A*
	Autosomal recessive	*ADSL*; *ALDH7A1*; *AMT*; *ASAH1*; *ASNS*; *CPLX1*; *CUBN*; *DOCK7*; *EPG5*; *GRM7*; *MBOAT7*; *MIPEP*; *NRROS*; *NRXN1*; *PLEKHG2*; *PLPBP*; *PNPO*; *RARS2*; *RYR3*; *SLC2A1*; *TBC1D24*; *TBCD*; *TNK2*; *UGP2*; *VRK2*; *WWOX*
	X-linked	*ARX*; *ALG13*; *CLCN4*; *CDKL5*; *DCX*; *GABRE*; *HUWE1*; *IQSEC2*; *MECP2*; *NONO*
Trinucleotide repeat disorders		*ARX*; *ATXN2*
Mitochondrial disorders		*MT-ND1*, *MT-ND5*, *POLG*, *SUCLA2*
Candidate genes		*ABCD1*; *ALPL*; *ASXL1*; *ATP2A2*; *CACNA1C*; *CD99L2*; *CLCN6*; *COL4A1*; *CYF1P1*; *CREBBP*; *DYNC1I1*; *GPT2*; *HDAC8*; *MED12*; *MED25*; *MYO18A*; *NDUFA10*; *PEMT*; *RALGAPB*; *SHROOM4*; *SOX5*; *STRADA*; *TAF1*; *TCF4*; *TCF20*

## Data Availability

No new data were created or analyzed in this study. Data sharing is not applicable to this article.

## References

[1] Zuberi S.M., Wirrell E., Yozawitz E., Wilmshurst J.M., Specchio N., Riney K., Pressler R., Auvin S., Samia P., Hirsh E. (2022). ILAE classification and definition of epilepsy syndromes with onset in neonates and infants: Position statement by the ILAE Task Force on Nosology and Definitions. Epilepsia.

[2] Fukuyama Y. (2001). History of clinical identification of West syndrome–in quest after the classic. Brain Dev..

[3] O’Callaghan F.J., Lux A.L., Darke K., Edwards S.W., Hancock E., Johnson A.L., Kennedy C., Newton R.W., Verity C.M., Osborne J.P. (2011). The effect of lead time to treatment and of age of onset on developmental outcome at 4 years in infantile spasms: Evidence from the United Kingdom Infantile Spasms Study. Epilepsia.

[4] Symonds J.D., Elliott K.S., Shetty J., Armstrong M., Brunklaus A., Cutcutache I., Diver L.A., Dorris L., Gardiner S., Jollands A. (2021). Early childhood epilepsies: Epidemiology, classification, aetiology, and socio-economic determinants. Brain.

[5] Pavone P., Striano P., Falsaperla R., Pavone L., Ruggieri M. (2014). Infantile spasms syndrome, West syndrome and related phenotypes: What we know in 2013. Brain Dev..

[6] Riikonen R. (2020). Infantile spasms: Outcome in clinical studies. Pediatr. Neurol..

[7] Specchio N., Wirrell E.C., Scheffer I.E., Nabbout R., Riney K., Samia P., Guerreiro M., Gwer S., Zuberi S., Wilmhurst J.M. (2022). International League Against Epilepsy classification and definition of epilepsy syndromes with onset in childhood: Position paper by the ILAE Task Force on Nosology and Definitions. Epilepsia.

[8] Lux A.L., Edwards S.W., Hancock E., Johnson A.L., Kennedy C.R., Newton R.W., O’Callaghan F.J.K., Verity C.M., Osborne J.P. (2005). The United Kingdom Infantile Spasms Study (UKISS) comparing hormone treatment with vigabatrin on developmental and epilepsy outcomes to age 14 months: A multicentre randomized trial. Lancet Neurol..

[9] Darke K., Edwards S.W., Hancock E., Johnson A.L., Kennedy C.R., Lux A.L., Newton R.W., O’Callaghan F.J.K., Verity C.M., Osborne J.P. (2010). Developmental and epilepsy outcomes at age 4 years in the UKISS trial comparing hormonal treatments to vigabatrin for infantile spasms: A multi-centre randomized trial. Arch. Dis. Child..

[10] Peng P., Kessi M., Mao L., He F., Zhang C., Chen C., Pang N., Yin F., Pan P., Peng J. (2022). Etiologic classification of 541 infantile spasms cases: A cohort study. Front. Pediatr..

[11] Michaud J.L., Lachance M., Hamdan F.F., Carmant L., Lortie A., Diadori P., Major P., Meijer I.A., Lemyre E., Cossette P. (2014). The genetic landscape of infantile spasms. Hum. Mol. Genet..

[12] Liu L.Y., Lu Q., Wang Q.H., Wang Y.Y., Zhang B., Zou L.P. (2022). Diagnostic yield of a multi-strategy genetic testing procedure in a nationwide cohort of 728 patients with infantile spasms in China. Seizure.

[13] Nagarajan B., Gowda V.K., Yoganathan S., Sharawat I.K., Srivastava K., Vora N., Badheka R., Danda S., Kalane S., Kaur K. (2023). Landscape of genetic infantile epileptic spasms syndrome—A multicenter cohort of 124 children from India. Epilepsia Open.

[14] Oliver K.L., Scheffer I.E., Bennett M.F., Grinton B.E., Bahlo M., Berkovic S.F. (2023). Genes4Epilepsy: An epilepsy gene resource. Epilepsia.

[15] McTague A., Howell K.B., Cross J.H., Kurian M.A., Scheffer I.E. (2016). The genetic landscape of the epileptic encephalopathies of infancy and childhood. Lancet Neurol..

[16] Menezes L.F.S., Sabiá Júnior E.F., Tibery D.V., Carneiro L.D.A., Schwartz E.F. (2020). Epilepsy-related voltage-gated sodium channelopathies: A review. Front. Pharmacol..

[17] Ng A.C.H., Choudhary A., Barrett K.T., Gavrilovici C., Scantlebury M.H. (2023). Mechanisms of Infantile Epileptic Spasms Syndrome: What have we learned from animal models?. Epilepsia.

[18] Appenzeller S., Balling R., Barisic N., Baulac S., Caglayan H., Craiu D., De Jonghe P., Depienne C., Dimova P., Djemie T. (2014). De novo mutations in synaptic transmission genes including DNM1 cause epileptic encephalopathies. Am. J. Hum. Genet..

[19] Paciorkowski A.R., Thio L.L., Dobyns W.B. (2011). Genetic and biologic classification of infantile spasms. Pediatr. Neurol..

[20] Stafstrom C.E. (2009). Infantile spasms: A critical review of emerging animal models. Epilepsy Curr..

[21] Stafstrom C.E., Holmes G.L. (2002). Infantile spasms: Criteria for an animal model. Int. Rev. Neurobiol..

[22] Dulla C.G. (2018). Utilizing animal models of infantile spasms. Epilepsy Curr..

[23] Galanopoulou A.S., Moshé S.L. (2015). Pathogenesis and new candidate treatments for infantile spasms and early life epileptic encephalopathies: A view from preclinical studies. Neurobiol. Dis..

[24] Velíšek L., Jehle K., Asche S., Velíšková J. (2007). Model of infantile spasms induced by N-methyl-D-aspartic acid in prenatally impaired brain. Ann. Neurol. Off. J. Am. Neurol. Assoc. Child Neurol. Soc..

[25] Mareš P., Velišek L. (1992). N-methyl-D-aspartate (NMDA)-induced seizures in developing rats. Dev. Brain Res..

[26] Shi X.Y., Yang X.F., Tomonoh Y., Hu L.Y., Ju J., Hirose S., Zou L.P. (2015). Development of a mouse model of infantile spasms induced by N-methyl-D-aspartate. Epilepsy Res..

[27] Kabova R., Veresova S., Velisek L. (1997). West syndrome model: Seek and you will find. Sb. Lek..

[28] Lemke J.R., Hendrickx R., Geider K., Laube B., Schwake M., Harvey R.J., Weckhuysen S. (2014). GRIN2B mutations in West syndrome and intellectual disability with focal epilepsy. Ann. Neurol..

[29] Chachua T., Yum M.S., Velíšková J., Velíšek L. (2011). Validation of the rat model of cryptogenic infantile spasms. Epilepsia.

[30] Yum M.S., Chachua T., Velíšková J., Velíšek L. (2012). Prenatal stress promotes development of spasms in infant rats. Epilepsia.

[31] Wang J., Wang J., Zhang Y., Yang G., Zhou W.J., Shang A.J., Zou L.P. (2012). Proteomic analysis of adrenocorticotropic hormone treatment of an infantile spasm model induced by N-methyl-D-aspartic acid and prenatal stress. PLoS ONE.

[32] Shi X.Y., Zou L.P., Yang G., Ding Y.X. (2012). Prenatal stress exposure hypothesis for infantile spasms. Med. Hypotheses.

[33] Palo J., Savolainen H. (1974). The effect of high doses of synthetic ACTH on rat brain. Brain Res..

[34] Holmes G.L., Weber D.A. (1986). Effects of ACTH on seizure susceptibility in the developing brain. Ann. Neurol. Off. J. Am. Neurol. Assoc. Child Neurol. Soc..

[35] Huttenlocher P.R. (1974). Dendritic development in neocortex of children with mental defect and infantile spasms. Neurology.

[36] Riikonen R. (2023). Biochemical mechanisms in pathogenesis of infantile epileptic spasm syndrome. Seizure.

[37] Korhonen L., Riikonen R., Nawa H., Lindholm D. (1998). Brain derived neurotrophic factor is increased in cerebrospinal fluid of children suffering from asphyxia. Neurosci. Lett..

[38] Riikonen R.S., Korhonen L.T., Lindholm D.B. (1999). Cerebrospinal nerve growth factor—A marker of asphyxia?. Pediatr. Neurol..

[39] Levi-Montalcini R., Dal Toso R., della Valle F., Skaper S.D., Leon A. (1995). Update of the NGF saga. J. Neurol. Sci..

[40] Riikonen R., Kokki H. (2019). CSF nerve growth factor (β-NGF) is increased but CSF insulin-like growth factor-(IGF-1) is normal in children with tuberous sclerosis and infantile spasms. Eur. J. Paediatr. Neurol..

[41] Aloe L., Rocco M.L., Bianchi P., Manni L. (2012). Nerve growth factor: From the early discoveries to the potential clinical use. J. Transl. Med..

[42] Riikonen R.S., Jääskeläinen J., Turpeinen U. (2010). Insulin-like growth factor-1 is associated with cognitive outcome in infantile spasms. Epilepsia.

[43] Joshi K., Shen L., Michaeli A., Salter M., Thibault-Messier G., Hashmi S., Eubanks J.H., Snead O.C. (2016). Infantile spasms in Down syndrome: Rescue by knockdown of the GIRK2 channel. Ann. Neurol..

[44] Blichowski M., Shephard A., Armstrong J., Shen L., Cortez M.A., Eubanks J.H., Snead O.C. (2015). The GIRK 2 subunit is involved in IS-like seizures induced by GABA B receptor agonists. Epilepsia.

[45] Peerboom C., Wierenga C.J. (2021). The postnatal GABA shift: A developmental perspective. Neurosci. Biobehav. Rev..

[46] Crino P.B. (2009). Focal brain malformations: Seizures, signaling, sequencing. Epilepsia.

[47] Jansen L.A., Peugh L.D., Ojemann J.G. (2008). GABAA receptor properties in catastrophic infantile epilepsy. Epilepsy Res..

[48] Monfrini E., Borellini L., Zirone E., Yahya V., Mauri E., Molisso M.T., Mameli F., Ruggiero F., Comi G.C., Barbieri S. (2023). GABRB1-related early onset developmental and epileptic encephalopathy: Clinical trajectory and novel de novo mutation. Epileptic Disord..

[49] Farnaes L., Nahas S.A., Chowdhury S., Nelson J., Batalov S., Dimmock D.M., Kingsmore S.F., RCIGM Investigators (2017). Rapid whole-genome sequencing identifies a novel GABRA1 variant associated with West syndrome. Mol. Case Stud..

[50] Kodera H., Ohba C., Kato M., Maeda T., Araki K., Tajima D., Matsuo M., Hino-Fukuyo N., Kohashi K., Ishiyama A. (2016). De novo GABRA1 mutations in Ohtahara and West syndromes. Epilepsia.

[51] Janve V.S., Hernandez C.C., Verdier K.M., Hu N., Macdonald R.L. (2016). Epileptic encephalopathy de novo GABRB mutations impair γ-aminobutyric acid type A receptor function. Ann. Neurol..

[52] Lee C.L., Frost J.D., Swann J.W., Hrachovy R.A. (2008). A new animal model of infantile spasms with unprovoked persistent seizures. Epilepsia.

[53] Frost J.D., Le J.T., Lee C.L., Ballester-Rosado C., Hrachovy R.A., Swann J.W. (2015). Vigabatrin therapy implicates neocortical high frequency oscillations in an animal model of infantile spasms. Neurobiol. Dis..

[54] Friocourt G., Parnavelas J.G. (2010). Mutations in ARX result in several defects involving GABAergic neurons. Front. Cell. Neurosci..

[55] Price M.G., Yoo J.W., Burgess D.L., Deng F., Hrachovy R.A., Frost J.D., Noebels J.L. (2009). A triplet repeat expansion genetic mouse model of infantile spasms syndrome, Arx (GCG) 10 + 7, with interneuronopathy, spasms in infancy, persistent seizures, and adult cognitive and behavioral impairment. J. Neurosci..

[56] Marsh E., Fulp C., Gomez E., Nasrallah I., Minarcik J., Sudi J., Christian S.L., Mancini G., Labosky P., Dobyns W. (2009). Targeted loss of Arx results in a developmental epilepsy mouse model and recapitulates the human phenotype in heterozygous females. Brain.

[57] Simonet J.C., Sunnen C.N., Wu J., Golden J.A., Marsh E.D. (2015). Conditional loss of Arx from the developing dorsal telencephalon results in behavioral phenotypes resembling mild human ARX mutations. Cereb. Cortex.

[58] Scantlebury M.H., Galanopoulou A.S., Chudomelova L., Raffo E., Betancourth D., Moshé S.L. (2010). A model of symptomatic infantile spasms syndrome. Neurobiol. Dis..

[59] Langlais P.J., Wardlow M.L., Yamamoto H. (1991). Changes in CSF neurotransmitters in infantile spasms. Pediatr. Neurol..

[60] Galanopoulou A.S., Mowrey W.B., Liu W., Li Q., Shandra O., Moshé S.L. (2017). Preclinical screening for treatments for infantile spasms in the multiple hit rat model of infantile spasms: An update. Neurochem. Res..

[61] Gygax M.J., Klein B.D., White H.S., Kim M., Galanopoulou A.S. (2014). Efficacy and tolerability of the galanin analog NAX 5055 in the multiple-hit rat model of symptomatic infantile spasms. Epilepsy Res..

[62] Berg A.T., Chakravorty S., Koh S., Grinspan Z.M., Shellhaas R.A., Saneto R.P., Wirrell E.C., Coryell J., Chu C.J., Mytinger J.R. (2018). Why West? Comparisons of clinical, genetic and molecular features of infants with and without spasms. PLoS ONE.

[63] Frost J.D., Hrachovy R.A. (2005). Pathogenesis of infantile spasms: A model based on developmental desynchronization. J. Clin. Neurophysiol..

[64] Arya R., Kabra M., Gulati S. (2011). Epilepsy in children with Down syndrome. Epileptic Disord..

[65] Kats D.J., Roche K.J., Skotko B.G. (2020). Epileptic spasms in individuals with Down syndrome: A review of the current literature. Epilepsia Open.

[66] Harvey S., Allen N.M., King M.D., Lynch B., Lynch S.A., O’Regan M., O’Rourke D., Shahwan A., Webb D., Gorman K.M. (2022). Response to treatment and outcomes of infantile spasms in Down syndrome. Dev. Med. Child Neurol..

[67] Inoue H., Orita T., Matsushige T., Hasegawa S., Ichiyama T. (2012). Klinefelter’s syndrome complicated with West syndrome in a 4-month-old boy. Brain Dev..

[68] Jaspersen S.L., Bruns D.A., Candee M.S., Battaglia A., Carey J.C., Fishler K.P. (2023). Seizures in trisomy 18: Prevalence, description, and treatment. Am. J. Med. Genet. Part A.

[69] Toyo-Oka K., Shionoya A., Gambello M.J., Cardoso C., Leventer R., Ward H.L., Wynshaw-Boris A. (2003). 14-3-3ε is important for neuronal migration by binding to NUDEL: A molecular explanation for Miller–Dieker syndrome. Nat. Genet..

[70] Demarest S., Calhoun J., Eschbach K., Yu H.C., Mirsky D., Angione K., Shaikh T.H., Carvill G.L., Benke T.A., WES Support Group (2022). Whole-exome sequencing and adrenocorticotropic hormone therapy in individuals with infantile spasms. Dev. Med. Child Neurol..

[71] Palmer E.E., Sachdev R., Macintosh R., Melo U.S., Mundlos S., Righetti S., Kandula T., Minoche A.E., Puttick C., Gayevskiy V. (2021). Diagnostic yield of whole genome sequencing after nondiagnostic exome sequencing or gene panel in developmental and epileptic encephalopathies. Neurology.

[72] Liu L., Liu F., Wang Q., Xie H., Li Z., Lu Q., Wang Y., Zhang M., Zhang Y., Picker J. (2021). Confirming the contribution and genetic spectrum of de novo mutation in infantile spasms: Evidence from a Chinese cohort. Mol. Genet. Genom. Med..

[73] Duc N.M., Thu N.T.M., Bui C.B., Hoa G., Hieu N.L.T. (2023). Genotype and phenotype characteristics of west syndrome in 20 Vietnamese children: Two novel variants detected by next-generation sequencing. Epilepsy Res..

[74] Essajee F., Urban M., Smit L., Wilmshurst J.M., Solomons R., van Toorn R., Moosa S. (2022). Utility of genetic testing in children with developmental and epileptic encephalopathy (DEE) at a tertiary hospital in South Africa: A prospective study. Seizure.

[75] Byrne S., Jansen L., U-King-Im J.M., Siddiqui A., Lidov H.G., Bodi I., Smith L., Mein R., Cullup T., Dionisi-Vici C. (2016). EPG5-related Vici syndrome: A paradigm of neurodevelopmental disorders with defective autophagy. Brain.

[76] Cohen A.L., Mulder B.P., Prohl A.K., Soussand L., Davis P., Kroeck M.R., McManus P., Gholipour A., Scherrer B., Bebin E.M. (2021). Tuberous Sclerosis Complex Autism Center of Excellence Network Study Group. Tuber locations associated with infantile spasms map to a common brain network. Ann. Neurol..

[77] Aronica E., Specchio N., Luinenburg M.J., Curatolo P. (2023). Epileptogenesis in tuberous sclerosis complex-related developmental and epileptic encephalopathy. Brain..

[78] Feng Y., Wei Z.H., Liu C., Li G.Y., Qiao X.Z., Gan Y.J., Qiao X.Z., Gan Y.J., Zhang C.C., Deng Y.C. (2022). Genetic variations in GABA metabolism and epilepsy. Seizure.

[79] Gjerulfsen C.E., Mieszczanek T.S., Johannesen K.M., Liao V.W., Chebib M., Nørby H.A., Gardella E., Rubboli G., Ahring P., Møller R.S. (2023). Vinpocetine improved neuropsychiatric and epileptic outcomes in a patient with a GABRA1 loss-of-function variant. Ann. Clin. Transl. Neurol..

[80] Billakota S., Andresen J.M., Gay B.C., Stewart G.R., Fedorov N.B., Gerlach A.C., Devinsky O. (2019). Personalized medicine: Vinpocetine to reverse effects of GABRB3 mutation. Epilepsia.

[81] Absalom N.L., Liao V.W., Kothur K., Indurthi D.C., Bennetts B., Troedson C., Mohammad S.S., Gupta S., McGregor I.S., Bowen M.T. (2020). Gain-of-function GABRB3 variants identified in vigabatrin-hypersensitive epileptic encephalopathies. Brain Commun..

[82] Verhage M., Sørensen J.B. (2020). SNAREopathies: Diversity in mechanisms and symptoms. Neuron.

[83] McLeod F., Dimtsi A., Marshall A.C., Lewis-Smith D., Thomas R., Clowry G.J., Trevelyan A.J. (2023). Altered synaptic connectivity in an in vitro human model of STXBP1 encephalopathy. Brain.

[84] Houtman S.J., Lammertse H.C., van Berkel A.A., Balagura G., Gardella E., Ramautar J.R., Reale C., Moller R.S., Zara F., Striano P. (2021). STXBP1 syndrome is characterized by inhibition-dominated dynamics of resting-state EEG. Front. Physiol..

[85] Freibauer A., Wohlleben M., Boelman C. (2023). STXBP1-Related Disorders: Clinical Presentation, Molecular Function, Treatment, and Future Directions. Genes.

[86] Guiberson N.G.L., Pineda A., Abramov D., Kharel P., Carnazza K.E., Wragg R.T., Dittman J.S., Burré J. (2018). Mechanism-based rescue of Munc18-1 dysfunction in varied encephalopathies by chemical chaperones. Nat. Commun..

[87] Zhu Y.C., Xiong Z.Q. (2019). Molecular and synaptic bases of CDKL5 disorder. Dev. Neurobiol..

[88] Leonard H., Downs J., Benke T.A., Swanson L., Olson H., Demarest S. (2022). CDKL5 deficiency disorder: Clinical features, diagnosis, and management. Lancet Neurol..

[89] Olson H.E., Amin S., Bahi-Buisson N., Devinsky O., Marsh E.D., Pestana-Knight E., Rajaraman R.R., Aimetti A.A., Rybak E., Kong F. (2023). Long-term treatment with ganaxolone for seizures associated with cyclin-dependent kinase-like 5 deficiency disorder: 2-year open-label extension follow-up. Epilepsia.

[90] Malik I., Kelley C.P., Wang E.T., Todd P.K. (2021). Molecular mechanisms underlying nucleotide repeat expansion disorders. Nat. Rev. Mol. Cell Biol..

[91] Guerrini R., Moro F., Kato M., Barkovich A.J., Shiihara T., McShane M.A., Hurst J., Loi M., Tohyama J., Norci V. (2007). Expansion of the first PolyA tract of ARX causes infantile spasms and status dystonicus. Neurology.

[92] Paciorkowski A.R., Shafrir Y., Hrivnak J., Patterson M.C., Tennison M.B., Clark H.B., Gomez C.M. (2011). Massive expansion of SCA2 with autonomic dysfunction, retinitis pigmentosa, and infantile spasms. Neurology.

[93] Syrbe S., Harms F.L., Parrini E., Montomoli M., Mütze U., Helbig K.L., Polster T., Albrecht B., Bernbeck U., van Binsbergen E. (2017). Delineating SPTAN1 associated phenotypes: From isolated epilepsy to encephalopathy with progressive brain atrophy. Brain.

[94] Tohyama J., Nakashima M., Nabatame S., Gaik-Siew C.N., Miyata R., Rener-Primec Z., Kato M., Matsumoto M., Saitsu H. (2015). SPTAN1 encephalopathy: Distinct phenotypes and genotypes. J. Hum. Genet..

[95] Tsuji M., Kuroki S., Maeda H., Yoshioka M., Maihara T., Fujii T., Ito M. (2003). Leigh syndrome associated with West syndrome. Brain Dev..

[96] Finsterer J., Mahjoub S.Z. (2012). Epilepsy in mitochondrial disorders. Seizure.

[97] Wray C.D., Friederich M.W., du Sart D., Pantaleo S., Smet J., Kucera C., Fenton F., Scharer G., Van Coster R., Van Hove J.L. (2013). A new mutation in MT-ND1 m. 3928G> C p. V208L causes Leigh disease with infantile spasms. Mitochondrion.

[98] Brennan G.P., Henshall D.C. (2020). MicroRNAs as regulators of brain function and targets for treatment of epilepsy. Nat. Rev. Neurol..

[99] Morrison-Levy N., Borlot F., Jain P., Whitney R. (2021). Early-onset developmental and epileptic encephalopathies of infancy: An overview of the genetic basis and clinical features. Pediatr. Neurol..

[100] Hixson L., Goel S., Schuber P., Faltas V., Lee J., Narayakkadan A., Leung H., Osborne J. (2015). An overview on prenatal screening for chromosomal aberrations. J. Lab. Autom..

[101] Belkadi A., Bolze A., Itan Y., Cobat A., Vincent Q.B., Antipenko A., Shang L., Boisson B., Casanova J.L., Abel L. (2015). Whole-genome sequencing is more powerful than whole-exome sequencing for detecting exome variants. Proc. Natl. Acad. Sci. USA.

[102] Ewans L.J., Minoche A.E., Schofield D., Shrestha R., Puttick C., Zhu Y., Drew A., Gayevskiy V., Elakis G., Walsh C. (2022). Whole exome and genome sequencing in mendelian disorders: A diagnostic and health economic analysis. Eur. J. Hum. Genet..

[103] Lee S., Jang S., Kim J.I., Chae J.H., Kim K.J., Lim B.C. (2022). Whole genomic approach in mutation discovery of infantile spasms patients. Front. Neurol..

[104] D’Gama A.M., Mulhern S., Sheidley B.R., Boodhoo F., Buts S., Chandler N.J., Cobb J., Curtis M., Higginbotham E.J., Holland J. (2023). Evaluation of the feasibility, diagnostic yield, and clinical utility of rapid genome sequencing in infantile epilepsy (Gene-STEPS): An international, multicentre, pilot cohort study. Lancet Neurol..

[105] Smith L., Malinowski J., Ceulemans S., Peck K., Walton N., Sheidley B.R., Lippa N. (2023). Genetic testing and counseling for the unexplained epilepsies: An evidence-based practice guideline of the National Society of Genetic Counselors. J. Genet. Couns..

[106] Howell K.B., Eggers S., Dalziel K., Riseley J., Mandelstam S., Myers C.T., McMahon J.M., Schneider A., Carvill G.L., Mefford H.C. (2018). A population-based cost-effectiveness study of early genetic testing in severe epilepsies of infancy. Epilepsia.

[107] Palmer E.E., Schofield D., Shrestha R., Kandula T., Macintosh R., Lawson J.A., Andrews I., Sampaio H., Johnson A.M., Farrar M.A. (2018). Integrating exome sequencing into a diagnostic pathway for epileptic encephalopathy: Evidence of clinical utility and cost effectiveness. Mol. Genet. Genom. Med..

[108] Sanchez Fernandez I., Loddenkemper T., Gaínza-Lein M., Sheidley B.R., Poduri A. (2019). Diagnostic yield of genetic tests in epilepsy: A meta-analysis and cost-effectiveness study. Neurology.

[109] Varesio C., Gana S., Asaro A., Ballante E., Cabini R.F., Tartara E., Bagnaschi M., Pasca L., Valente M., Orcesi S. (2021). Diagnostic yield and cost-effectiveness of “dynamic” exome analysis in epilepsy with neurodevelopmental disorders: A tertiary-center experience in northern Italy. Diagnostics.

[110] Jegathisawaran J., Tsiplova K., Hayeems R., Ungar W.J. (2020). Determining accurate costs for genomic sequencing technologies—A necessary prerequisite. J. Community Genet..

[111] Pavone P., Polizzi A., Marino S.D., Corsello G., Falsaperla R., Marino S., Ruggieri M. (2020). West syndrome: A comprehensive review. Neurol. Sci..

[112] Al-Shehhi W., Chau V., Boyd J., Snead C., Sharma R., Donner E., Go C., Jain P. (2022). Treatment with High-Dose Prednisolone in Vigabatrin-Refractory Infantile Spasms. Can. J. Neurol. Sci..

[113] Kuchenbuch M., Lo Barco T., Chemaly N., Chiron C., Nabbout R. (2023). Fifteen years of real-world data on the use of vigabatrin in individuals with infantile epileptic spasms syndrome. Epilepsia.

[114] Dzau W., Cheng S., Snell P., Fahey M., Scheffer I.E., Harvey A.S., Howell K.B. (2022). Response to sequential treatment with prednisolone and vigabatrin in infantile spasms. J. Paediatr. Child Health.

[115] Grinspan Z.M., Knupp K.G., Patel A.D., Yozawitz E.G., Wusthoff C.J., Wirrell E.C., Valencia I., Singhal N.S., Nordli D.R., Wirrell E.C. (2021). Comparative effectiveness of initial treatment for infantile spasms in a contemporary US cohort. Neurology.

[116] Whitney R., Jain P. (2022). Steroids in infantile spasms syndrome: Another trial, another drug, another dose, what’s next?. Ann. Indian Acad. Neurol..

[117] Gettings J.V., Shafi S., Boyd J., Snead O.C., Rutka J., Drake J., McCoy B., Jain P., Whitney R., Go C. (2023). The Epilepsy Surgery Experience in Children With Infantile Epileptic Spasms Syndrome at a Tertiary Care Center in Canada. J. Child Neurol..

[118] Specchio N., Pietrafusa N., Ferretti A., De Palma L., Santarone M.E., Pepi C., Trivisano M., Curatolo P. (2020). Treatment of infantile spasms: Why do we know so little?. Expert Rev. Neurother..

[119] Nouri M.N., Zak M., Jain P., Whitney R. (2022). Epilepsy management in tuberous sclerosis Complex: Existing and evolving therapies and future considerations. Pediatr. Neurol..

[120] Bebin E.M., Peters J.M., Porter B.E., McPherson T.O., O’Kelley S., Sahin M., Taub K.S., Rajaraman R., Randle S.C., McClintock W.M. (2023). Early Treatment with Vigabatrin Does Not Decrease Focal Seizures or Improve Cognition in Tuberous Sclerosis Complex: The PREVeNT Trial. Ann. Neurol..

[121] Kotulska K., Kwiatkowski D.J., Curatolo P., Weschke B., Riney K., Jansen F., Feucht M., Krsek P., Nabbout R., Jansen A.C. (2021). Prevention of epilepsy in infants with tuberous sclerosis complex in the EPISTOP trial. Ann. Neurol..

[122] Samueli S., Abraham K., Dressler A., Gröppel G., Mühlebner-Fahrngruber A., Scholl T., Kasprian G., Laccone F., Feucht M. (2016). Efficacy and safety of Everolimus in children with TSC-associated epilepsy–Pilot data from an open single-center prospective study. Orphanet J. Rare Dis..

[123] He W., Chen J., Wang Y.Y., Zhang M.N., Wang Q.H., Luo X.M., Chen X.Q., Zou L.P. (2020). Sirolimus improves seizure control in pediatric patients with tuberous sclerosis: A prospective cohort study. Seizure.

[124] Curatolo P., Nabbout R., Lagae L., Aronica E., Ferreira J.C., Feucht M., Hertzberg C., Jansen A.C., Jansen F., Kotulska K. (2018). Management of epilepsy associated with tuberous sclerosis complex: Updated clinical recommendations. Eur. J. Paediatr. Neurol..

[125] Velíšek L., Velíšková J. (2020). Modeling epileptic spasms during infancy: Are we heading for the treatment yet?. Pharmacol. Ther..

[126] Hack J.B., Horning K., Juroske Short D.M., Schreiber J.M., Watkins J.C., Hammer M.F. (2023). Distinguishing loss-of-function and gain-of-function SCN8A variants using a random forest classification model trained on clinical features. Neurol. Genet..

[127] Wolff M., Johannesen K.M., Hedrich U.B., Masnada S., Rubboli G., Gardella E., Lesca G., Ville D., Milh M., Villard L. (2017). Genetic and phenotypic heterogeneity suggest therapeutic implications in SCN2A-related disorders. Brain.

[128] Hussain S.A., Heesch J., Weng J., Rajaraman R.R., Numis A.L., Sankar R. (2021). Potential induction of epileptic spasms by nonselective voltage-gated sodium channel blockade: Interaction with etiology. Epilepsy Behav..

[129] Olivetti P.R., Maheshwari A., Noebels J.L. (2014). Neonatal estradiol stimulation prevents epilepsy in Arx model of X-linked infantile spasms syndrome. Sci. Transl. Med..

[130] Kerrigan J.F., Shields W.D., Nelson T.Y., Bluestone D.L., Dodson W.E., Bourgeois B.F., Pellock J.M., Morton L.D., Monaghan E.P. (2000). Ganaxolone for treating intractable infantile spasms: A multicenter, open-label, add-on trial. Epilepsy Res..

[131] Ho P.T., Clark I.M., Le L.T. (2022). MicroRNA-based diagnosis and therapy. Int. J. Mol. Sci..

[132] Mendell J.R., Al-Zaidy S., Shell R., Arnold W.D., Rodino-Klapac L.R., Prior T.W., Lowes L., Alfano L., Berry K., Church K. (2017). Single-dose gene-replacement therapy for spinal muscular atrophy. New Engl. J. Med..

[133] Mich J.K., Ryu J., Wei A.D., Gore B.B., Guo R., Bard A.M., Martinez R.A., Bishaw Y., Luber E., Santos L.M.O. (2023). AAV-mediated in-terneuron-specific gene replacement for Dravet syndrome. bioRxiv.

[134] Shaimardanova A.A., Chulpanova D.S., Mullagulova A.I., Afawi Z., Gamirova R.G., Solovyeva V.V., Rizvanov A.A. (2022). Gene and cell therapy for epilepsy: A mini review. Front. Mol. Neurosci..

[135] Goodspeed K., Liu J.S., Nye K.L., Prasad S., Sadhu C., Tavakkoli F., Bilder D.A., Minassian B.A., Bailey R.M. (2022). SLC13A5 deficiency disorder: From genetics to gene therapy. Genes.

[136] Johannesen K.M., Tümer Z., Weckhuysen S., Barakat T.S., Bayat A. (2023). Solving the unsolved genetic epilepsies: Current and future perspectives. Epilepsia.

[137] Wanigasinghe J., Sahu J.K., Madaan P., Fatema K., Linn K., Chand P., Poudel P., Hamed E., Mynak M.L., Hassan S. (2021). Classifying etiology of infantile spasms syndrome in resource-limited settings: A study from the South Asian region. Epilepsia Open.

[138] Knowles J.K., Helbig I., Metcalf C.S., Lubbers L.S., Isom L.L., Demarest S., Goldberg E.M., George A.L., Lerche H., Weckhuysen S. (2022). Precision medicine for genetic epilepsy on the horizon: Recent advances, present challenges, and suggestions for continued progress. Epilepsia.

